# Systematic review of mental health disorders and intimate partner violence victimisation among military populations

**DOI:** 10.1007/s00127-017-1423-8

**Published:** 2017-07-26

**Authors:** Katherine Sparrow, Jamie Kwan, Louise Howard, Nicola Fear, Deirdre MacManus

**Affiliations:** 1 Forensic and Neurodevelopmental Sciences Department, Institute of Psychiatry Psychology and Neuroscience, King’s College London, PO23, 16 De Crespigny Park, London, SE5 8AF UK; 2 Department of Psychological Medicine, Weston Education Centre, 10 Cutcombe Road, London, SE5 9RJ UK; 3 David Goldberg Centre, Institute of Psychiatry Psychology and Neuroscience, King’s College London, 16 De Crespigny Park, London, SE5 8AF UK; 4 King’s Centre for Military Health Research, King’s College London, Weston Education Centre, 10 Cutcombe Road, London, SE5 9RJ UK

**Keywords:** Military, Mental disorder, Intimate partner violence, Review

## Abstract

**Purpose:**

There is growing awareness of the problem of intimate partner violence (IPV) among military populations. IPV victimisation has been shown to be associated with mental disorder. A better understanding of the link between IPV and mental disorder is needed to inform service development to meet the needs of military families. We aimed to systematically review the literature on the association between IPV victimisation and mental health disorders among military personnel.

**Methods:**

Searches of four electronic databases (Embase, Medline, PsycINFO, and Web of Science) were supplemented by reference list screening. Heterogeneity among studies precluded a meta-analysis.

**Results:**

Thirteen studies were included. There was stronger evidence for an association between IPV and depression/alcohol problems than between IPV and PTSD. An association between IPV and mental health problems was more frequently found among veterans compared to active duty personnel. However, the link between IPV and alcohol misuse was more consistently found among active duty samples. Finally, among active duty personnel psychological IPV was more consistently associated with depression/alcohol problems than physical/sexual IPV. The review highlighted the lack of research on male IPV victimisation in the military.

**Conclusions:**

There is evidence that the burden of mental health need may be significant among military personnel who are victims of IPV. The influence of attitudes towards gender in the military on research in this area is discussed. Further research is needed to inform development of services and policy to reduce IPV victimisation and the mental health consequences among military personnel.

**Electronic supplementary material:**

The online version of this article (doi:10.1007/s00127-017-1423-8) contains supplementary material, which is available to authorized users.

## Introduction

Intimate partner violence (IPV) is a serious, preventable public health problem that occurs in all settings and among all religious, cultural, and socioeconomic groups [1]. IPV includes physical and sexual violence, stalking, and psychological aggression (including coercive controlling behaviour) by a current or former intimate partner [2]. It has been estimated to account for up to 7% of the overall burden of disease among women, primarily due to mental health impairment [3, 4]. IPV research among military populations lags behind that in civilian populations [5–7]. The well-publicised murders of the wives of four American Fort Bragg soldiers in the space of 6 weeks in 2002 [8] led to increased awareness within international Armed Forces communities of the reality of IPV among military couples. Military couples^1^ are exposed to some unique stressors which have been shown to impact negatively on relationships and some of which have been associated with increased risk of IPV [9], including operational deployments and deployment related injuries [10–13], combat exposure [13, 14] and service-related mental health and behavioural problems [15, 16], frequent relocation, and familial separation [17–19]. The experience of military service and the consequences of some of these stressors can continue to impact on relationships long after the serving person has left the Armed Forces [20–29]. Transitioning out of the military is also associated with a range of additional psychosocial stressors [30–35] and veterans have been shown to report high levels of some mental health problems [36–41]. It should, therefore, not be assumed that the correlates of IPV are consistent across civilian and military couples [42, 43] and they may even differ between military couples with an active serving partner and those with a partner who is a veteran [5, 41].

There is a growing body of research on IPV victimisation among military populations, though these studies are very heterogeneous in terms of samples, method of measurement of IPV, and definitions of different types of IPV. To our knowledge, no UK studies exist. Studies in the US have found high levels of IPV victimisation among military personnel, both male and female [44–49], with conflicting conclusions on whether IPV victimisation is higher among males or females, depending on the severity of violence measured [50, 51]. Many of the other risk factors for IPV victimisation in the general population have been found to be important among military populations also such as age [52–55], social class [56], and level of education [53, 57], though findings are not consistent [44, 52, 56–59]. It has been suggested by some studies that IPV may be more prevalent among military than civilian populations [46, 60, 61], though this has also not been a universal finding [62].

There is a large body of literature which has established the link between IPV victimisation and mental disorder in the general population. Research has focused on depression, PTSD, anxiety, eating disorders, substance misuse, and chronic mental illness more broadly, with the most consistent evidence highlighting a link between IPV and depression, followed by PTSD and anxiety disorders [63–72]. There is evidence to suggest a causal association between IPV and mental disorders in both directions: IPV can lead to negative mental health outcomes, and mental health problems can render a person more vulnerable to experiencing IPV [73]. A recent systematic review found evidence for an association between IPV perpetration and mental disorders among military populations [74]. A number of studies have also explored the association between IPV victimisation and mental disorders among military personnel. The methodological rigour and hence the findings of these studies have varied greatly. A systematic review of such studies is needed to gain a better understanding of the link between IPV victimisation and mental health problems, such as depression, PTSD, anxiety disorders, and substance misuse, among military personnel, to inform the development of services to meet the needs of military families.

The aim of this study was, therefore, to systematically review extant studies to summarise the literature exploring IPV victimisation and specific mental health problems among male and female military personnel (both serving and ex-serving).

## Methods

A literature search was undertaken for studies examining mental health problems associated with IPV victimisation among military populations. Searches of the following electronic databases were carried out: Embase, Medline, PsycINFO, and Web of Science. The search terms and combinations used were identical for all four databases. Search results were limited to papers published in English. In addition to searching bibliographic databases, the reference lists of all relevant papers and reviews were searched. Authors were contacted to request raw data where necessary. This review followed PRISMA reporting guidelines and the protocol is registered with PROSPERO: registration CRD42016044119.

Studies were eligible for inclusion if they: (1) involved male and/or female serving or ex-serving military personnel; (2) reported the risk of IPV victimisation among those with and without mental disorder or vice versa, and/or a measure of association between IPV and mental disorder; (3) measured IPV using a validated tool or adapted question(s); (4) measured mental health using a validated diagnostic or screening tool, e.g., the PTSD checklist (PCL), or the Alcohol Use Disorders Identification Test (AUDIT); (5) presented the results of peer reviewed research based on any quantitative study design capable of providing the data listed above; and (6) had a sample size of over 100 participants. IPV was defined as “any incident of threatening behaviour, violence or abuse (psychological, physical, sexual, financial, or emotional) between adults who are or have been intimate partners regardless of gender or sexuality” [75]. Mental disorders included schizophrenia and psychotic disorders, mood disorders, neurotic and stress-related disorders (including anxiety disorders and post-traumatic stress disorder), eating disorders, and mental and behavioural disorders related to alcohol or substance misuse. Titles and abstracts were screened against the inclusion criteria. The full texts of potentially eligible studies were then reviewed. Quality appraisal of the included studies was conducted independently by two reviewers using a checklist adapted from validated tools [76–80] (see supplementary information). Agreement for the overall quality appraisal scores for the 13 studies was calculated using the Kappa statistic (Kappa = 0.74). A third, more senior, reviewer was consulted in the instance of any scoring discrepancies. Studies that scored 50% or higher on criteria relating to selection bias were categorised as high quality. This review focused on studies which allowed the estimation of risk of IPV among individuals with and without mental health disorder. The 50% criterion was selected to identify studies with a lower risk of selection bias and on whose findings greater weight could be placed. Qualitative and quantitative data were extracted from included studies, including information on study design, sample characteristics, and measurement tools used, as well as data on the risk of IPV victimisation and mental disorder. Data were extracted separately for men and women, where possible.

**Fig. 1:**
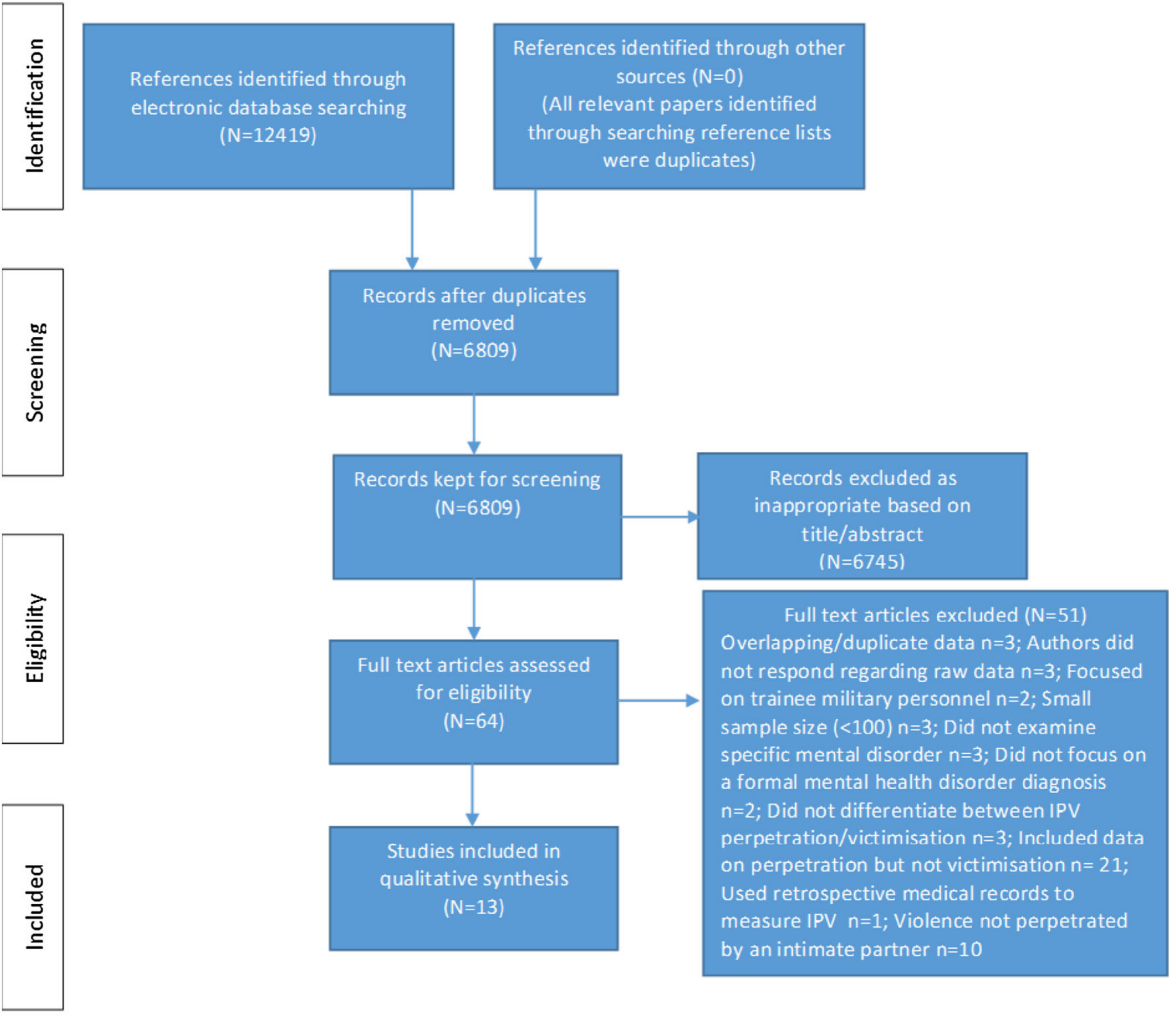
Flow of information through the phases of the systematic literature search

Figure 1 describes the study selection process. Literature searches yielded 6809 unique references; 6745 were excluded following title and abstract screening and a further 51 were excluded following full-text screening. The remaining 13 papers were included in this review. All 13 papers were identified through searches of electronic databases. References identified through other sources (i.e., screening the reference lists of included studies) were all duplicates. The 13 papers reported on a combined sample of 55,883 participants.

Heterogeneity among the studies in this review (primarily regarding the timing and type of IPV studied) precluded a meta-analysis.

## Results

### Key features of included studies

The key characteristics of the included studies are summarised in Table 1. All studies were conducted in high-income countries, with two conducted in Canada [44, 45], and the other 11 conducted in the USA. Five of the 13 studies were conducted in clinical settings [52, 81–84] and eight in non-clinical settings [44, 45, 47, 48, 50, 54, 58, 85]. Five of the 13 studies were categorised as high quality (i.e., scoring 50% or higher for selection bias) [44, 45, 47, 48, 50].

**Table 1:** Key features of included studies

	Total (*n* = 13)
Study design	
Cross-sectional	13
Gender^a^	
No. of papers reporting on male victims	3 [6, 8, 9]
No. of papers reporting on female victims	9 [7, 8, 33–39]
Papers reporting on male and female victims together—unable to get separate data	2 [10, 11]
Setting	
Clinical setting	5 [33–36, 39]
General military setting	6 [8–11, 38, 39]
Community	2 [6, 7]
Sample	
Air force (serving)	1 [8]
Army (serving)	2 [8, 9]
Veterans	7 [6, 7, 33–37]
Armed forces (all services—serving)	3 [10, 11, 39]
Timing of IPV	
Past-year	4 [8, 9, 36, 38]
Lifetime	6 [6, 7, 33–35, 39]
Over course of current relationship	2 [10, 11]
During military service	1 [37]
Type of IPV^a^	
Physical	5 [9, 10, 33–35]
Sexual	2 [34, 37]
Psychological/emotional	5 [6, 8–10, 34]
Any IPV—(varying definitions)	6 [6, 7, 11, 36, 38, 39]
IPV measure	
Validated tool	6 [8, 10, 11, 34, 36, 38]
Modified version of validated tool	4 [9, 33, 35, 39]
Objective IPV question	3 [6, 7, 37]
Quality appraisal score	
Low quality	4 [33–36]
Medium quality	4 [9, 37–39]
High quality	5 [6–8, 10, 11]

^a^As categories (Gender and Type of IPV) are not mutually exclusive, totals may exceed 13

As shown in Table 1, nine studies reported on female victims, three studies reported on male victims, and two studies included both males and females, but did not report mental health outcomes by gender. Seven studies explored the link between IPV and depression, five studies focused on IPV and PTSD, and five on IPV and alcohol problems (see Table 2). Six of the 13 studies utilised validated measures of IPV such as the Conflict Tactics Scale (CTS) or the Abuse Assessment Screen to measure domestic violence. Four studies used adapted versions of validated tools [54, 58, 81, 83]. The remaining three studies measured IPV using an objective question [47, 48, 52] (see Table 3).

**Table 2:** Associations between IPV victimisation and mental disorders

	Cerulli, 2014	Dobie, 2004	Dichter, 2011	Dichter, 2014	Foran, 2014^a^	Foran, 2014^a^	Iverson, 2015	Mercado, 2015	O’Campo, 2006	Rosen, 2002	Skomorovsky, 2015	Zamorski, 2013
Males/females/both	M	F	F	F	F	M	F	F	F	M	B	B
Mental disorder												
Depression	+		+				+		0	+	+	+
PTSD		+					+	+	0			0
Alcohol use problems				+	+	+	0			+		+
Mental health multi-morbidity							+					

Studies that explored IPV and mental health disorders but did not conduct a measure of association are not included in the table

*Blank* Variable not analysed (or not measured using validated tool), **+** Positive association found, **−** Negative association found, *0* No significant association found, *M* Male sample, *F* Female sample, *B* Both males and females in sample

^a^This study reported separate data for males and females

**Table 3:** Characteristics of included studies—partner violence victimisation

First author, year	Sample (size, type and gender distribution)	Method (measure of IPV and timing/type of IPV)	Prevalence of IPV	Measure of mental disorder	Mental health problem by IPV status	Measure of association between IPV and mental disorder	Quality appraisal
Depression							
Cerulli, 2014	4356 randomly selected male veterans completed nation-wide telephone survey of noninstitutionalized adults (age ≥18). Sample stratified by veteran status	Any (lifetime) IPV: actual or threatened physical violence or unwanted sex—measured using objective IPV question	Lifetime IPV prevalence: (413/4356) 9.5%	Patient Health Questionnaire-8, cutoff ≥10, (measures symptoms experienced over last 2 weeks) Prevalence of depression: 269/4356 (6.2%)	Prevalence of depression: Among those who report IPV: 71/413 = 17.2% Among those who do not report IPV: 342/3943 = 8.7%	aOR 2.63, 95% CI 1.49, 4.65	Total score: 31/42 Selection score: 7/14 Measurement score: 13/14
Dichter, 2011	503 randomly selected female veterans completed nation-wide telephone survey of noninstitutionalized adults (age ≥18)	Any (lifetime) IPV: actual or threatened physical violence or unwanted sex—measured using objective IPV question	Lifetime IPV prevalence: 167/476^a^ (35.1%)	Patient Health Questionnaire-8, cutoff ≥10 (measures symptoms over last 2 weeks) Prevalence of depression: 47/503 (9.3%)	Prevalence of depression: Among those who report IPV: 27/167 = 16.2% Among those who do not report IPV: 31/309 = 10%	aOR, 3.8 95% CI 3.2–4.5	Total score: 29/42 Selection score: 7/14 Measurement score: 12/14
Forgey, 2010	All enlisted females married to civilian spouses at large Army installation included in sample (*n* = 248)	Any past-year (physical/sexual/psychological) IPV measured using Conflict Tactics Scale-2 (CTS-2)	Past-year unilateral IPV prevalence, male perpetrator: 42/248 (16.9%)	Zung Self Rating Depression Scale, measures current symptoms Mean depression score (range 0–0.80): 0.49	Depression scores: Among those who report IPV: M = 0.51, SD = 0.12 (Violence Group 6^b^) Among those who do not report IPV: M = 0.44, SD = 0.10 (Group 1—no violence) Statistically significant difference between groups (*p* < 0.001)	No statistical measure of association between depression and IPV conducted	Total score: 22/42 Selection score: 6/14 Measurement score: 5/14
Iverson, 2015	160 randomly selected New England female VA patients with intimate relationship in past year	Any past-year IPV (physical or psychological) measured using Hurt, Insult, Threaten, Scream (HITS) tool	Past-year IPV prevalence: 58/160 (36.3%)	Center for Epidemiological Studies Depression Scale—CES-D, cutoff ≥16 (symptoms over past 2 weeks) Prevalence of depression: 78/158 (49.4%)	Prevalence of depression: Among those who report IPV: 39/58 = 67.2% Among those who do not report IPV: 19/102 = 18.6%	aOR 3.02, 95% CI 1.46–6.26	Total score: 31/42 Selection score: 5/14 Measurement score: 13/14
Mercado, 2015	369 randomly selected New England female VA patients	Sexual IPV during military service assessed using objective IPV question	Prevalence of sexual IPV during military service: 27/360^a^ (7.5%)	CES-D measures past-week depressive symptoms Adjusted mean depression score: 18.7	Depression scores: Among those who report sexual IPV: 19.7 (95% CI 14.4–25.0) Among those who do not report sexual IPV: 14.5 (95% CI 12.6–16.5) No significant difference between mean scores		Total score: 32/42 Selection score: 6/14 Measurement score: 13/14
O’Campo, 2006	616 randomly selected active duty tri-service females (Army, Air Force, Navy/Marines) in greater Washington, DC	Lifetime physical and/or sexual IPV measured using modified version of Abuse Assessment Screen	Lifetime physical or sexual abuse prevalence: 184/616 = 30% (25.4%)^d^	Brief Symptom Inventory, cutoff ≥1.11 (measures symptoms occurring in past week) Prevalence of depression: 69/468 (14.7%)	Prevalence of depression: Among those who reported IPV: 23/119 = 19.3% Among those who do not report IPV: 46/349 = 13.2%	aOR 1.67, 95% CI 0.83–3.36 NS	Total score: 33/42 Selection score: 6/14 Measurement score: 14/14
Rosen, 2002	488 (358 White males; 130 Black, males) married soldiers at Army post in Alaska. On average, approximately 60% of personnel from available units provided with opportunity to complete questionnaire	Any past-year physical, and any past-year psychological IPV measured using modified CTS (MCTS)	Prevalence of minor physical IPV: 114/488 (23.4%) Severe physical IPV: 78/488 (16%) Psychological IPV prevalence rate not reported	CES-D scale measures past-week depressive symptoms Mean depression score (range 0–3): 0.61	No data available	Severe physical IPV: *f* = 39.8, *p* < 0.01 Mild physical IPV: *f* = 11.0, *p* < 0.01 Psychological IPV: *f* = 5.4, *p* < 0.05	Total score: 25/42 Selection score: 6/14 Measurement score: 9/14
Skomorovsky, 2015	529 randomly selected Canadian Armed Forces members (males and females) at three military bases, in current intimate relationship. Note: males and females not studied separately due to low sample sizes	Physical IPV and emotional IPV over the course of the current relationship measured using Canadian General Social Survey	Emotional violence 138/529 (26.2%); physical violence 70/529 (13.2%)	CES-D scale measures past-week depressive symptoms Mean depression score (range 1–36): 15.2, SD 6.3	No data available	Multiple regression analyses: Emotional violence victimisation: β = 0.18 *p* < 0.01 Physical violence victimisation: β = 0.01 NS	Total score: 27/42 Selection score: 8/14 Measurement score: 7/14
Zamorski, 2013	1745 randomly selected (1017 males; 728 females) Regular active duty Canadian Armed Forces members in current relationship	Any IPV (physical/sexual/psychological—including financial abuse) experienced over the course of the current relationship measured using Canadian General Social Survey	Any physical and/or sexual IPV: both males and females 170/1745, 15.4%^d^ (95% CI 12.3–18.5) Any emotional and/or financial IPV: both males and females 387/1745, 25.1%^d^ (95% CI 21.6–28.7)	CIDI-SF, cutoff ≥5 (measures symptoms over previous 12 months) Prevalence of depression (males and females): 123^a^/1688 = 7.1% (95% CI 5.0–9.1)	Prevalence of depression: Among those who report physical and/or sexual IPV: 20/121^a^ = 26% (95% CI 10.6–41.4) Among those who do not report physical and/or sexual IPV: 138/1524 = 14.4%, (95% CI 11.2–17.6) Prevalence of depression: Among those who report emotional and/or financial IPV: 50/121 = 48.8% (95% CI 33.4–64.3) Among those who do not report emotional and/or financial IPV: 307/1524 = 22.5% (95% CI 18.9–26.0)	Physical and/or sexual IPV: aOR 1.54, 95% CI 0.55–4.33 NS Emotional/financial IPV: aOR 2.47, 95% CI 1.18–5.18	Total score: 37/42 Selection score: 11/14 Measurement score: 13/14
PTSD							
Dobie, 2004	1206 female VA patients in Washington (all patients receiving care between 1^st^ October 1996—1^st^ January 1998 mailed a survey)	Lifetime physical IPV: “At any time, has a partner ever hit, kicked, or otherwise physically hurt you?”	Lifetime prevalence: 432/1115^a^ (38.7%)	PCL-C, cutoff ≥50 (measures symptoms over past month) Prevalence of PTSD: 266/1206 (22.1%)	Prevalence of PTSD: Among those who report IPV: 139/432 = 32.2% Among those who do not report IPV: 101/683 = 14.8%	aOR 2.58 (95% CI 1.92–3.46)	Total score: 27/42 Selection score: 5/14 Measurement score: 5/14
Iverson, 2015	160 randomly selected New England female VA patients with intimate relationship in past year	Any past-year IPV (physical or psychological) measured using Hurt, Insult, Threaten, Scream (HITS) tool	Past-year IPV prevalence: 58/160 (36.3%)	PTSD Checklist, cutoff 50 (measures symptoms over previous month) Prevalence of PTSD: 44/156^a^ (28.2%)	Prevalence of PTSD: Among those who report IPV: 24/58 = 41.4% Among those who do not report IPV: 20/102 = 19.6%	aOR 2.35, 95% CI 1.08–5.08	Total score: 31/42 Selection score: 5/14 Measurement score: 13/14
Mercado 2015	369 randomly selected New England female VA patients	Sexual IPV during military service assessed using objective IPV question	Prevalence of sexual IPV during military service: 27/360^h^ (7.5%)	PTSD Checklist measures symptoms over previous month Mean PTSD score (range 17–85): 37.8	PTSD scores: Among those who report sexual IPV: 41.7 (95% CI 35.0–48.5) Among those who do not report sexual IPV: 29.7 (95% CI 27.2–32.3)	ANCOVA: *F*(2, 4255) = 12.27, *p* < 0.0001 Significantly higher levels of PTSD symptoms among women who had IPV-related MST compared to women with no MST history (adjusted mean PCL score: 41.7 vs. 29.7, respectively, *p* < 0.004) No significant difference in level of PTSD symptoms between women with IPV-related MST and women who had MST by a non-intimate partner (adjusted mean PCL score: 41.7 vs. 42.0, respectively, *p* = 1.0)	Total score: 32/42 Selection score: 6/14 Measurement score: 13/14
O’Campo, 2006	616 randomly selected active duty tri-service (Army, Air Force, Navy/Marines) females in greater Washington, DC	Lifetime physical and/or sexual IPV measured using modified version of Abuse Assessment Screen	Lifetime physical or sexual abuse prevalence: 119/468 = 25.4%^c^	Crime Related PTSD scale for Women. Presence of PTSD defined as reporting (a) at least one re-experiencing symptom, (b) at least three avoidance/numbing symptoms, (c) at least two symptoms of increased arousal Prevalence of PTSD: 12/468 (2.6%)	Prevalence of PTSD: Among those who report IPV: 6/119 = 5% Among those who do not report IPV: 6/349 = 1.7%	aOR = 2.92, 95% CI 0.88–9.63 NS	Total score: 33/42 Selection score: 6/14 Measurement score: 14/14
Zamorski, 2013	1745 randomly selected (1017 males; 728 females) Regular active duty Canadian Armed Forces members in current relationship	Any IPV (physical/sexual/psychological—including financial abuse) experienced over the course of the current relationship measured using Canadian General Social Survey	Any physical and/or sexual IPV: both males and females 170/1745, 15.4%^d^ (95% CI 12.3–18.5) Any emotional and/or financial IPV: both males and females 387/1745, 25.1%^d^ (95% CI 21.6–28.7)	Primary Care PTSD Screen, cutoff ≥3 (measures symptoms over past month) Prevalence of PTSD: 113^a^/1741, 7.3% (95% CI 5.0–9.5)	Prevalence of PTSD: Among those who report physical and/or sexual IPV: 21/109^a^ = 28% (95% CI 12.5–43.5) Among those who do not report physical and/or sexual IPV: 143/1585 = 14.2% (95% CI 11.1–17.3) Prevalence of PTSD: Among those who report emotional and/or financial IPV: 42/109 = 47.2% (95% CI 30.6–63.7) Among those who do not report emotional and/or financial IPV: 332/1583 = 23.2% (95% CI 19.6–26.7)	Physical and/or sexual IPV: aOR 1.89 (95% CI 0.77–4.65). NS Emotional and/or financial IPV: aOR 1.93 (95% CI 0.89–4.17). NS	Total score: 37/42 Selection score: 11/14 Measurement score: 13/14
Alcohol problems							
Chavez, 2012	2670 female VA patients. All female veterans receiving outpatient care from urban VA in western United States between October 1996 and January 1998 mailed a survey	Lifetime physical IPV: “At any time has a partner ever hit, kicked, or otherwise physically hurt you?”	Lifetime prevalence: 796/2528^e^ (31.5%)	AUDIT-C, cutoff ≥3 (measures symptoms over past year) Prevalence of alcohol problems: 644/2670 (24.1%)	Prevalence of alcohol problems: Among those who report IPV: (AUDIT-C score ≥3): 221/796 = 27.8% Among those who do not report IPV: (AUDIT-C score ≥3): 423/1732 = 24.4%	Lifetime domestic violence increased significantly with AUDIT-C scores of 5 or more *p* < 0.05 (No statistical measure of association between alcohol problems and IPV conducted in study)	Total score: 27/42 Selection score: 5/14 Measurement score: 8/14
Dichter, 2014	249 female VA patients receiving primary care services in 2012 in Philadelphia. Participants recruited via flyers/announcements in waiting room of Women’s Health Clinic	Any lifetime physical/sexual/ psychological IPV measured using CTS-2	Lifetime sexual IPV: 87/249 (35%) Lifetime physical IPV: 142/249 (57%) Lifetime psychological IPV: 204/249 (82%) Lifetime (any) IPV: 214/249 (85.9%)	CAGE scale, cutoff ≥2 (measures symptoms over lifetime) Prevalence of problem drinking: 52 (21%)	Prevalence of problem drinking: Among those who report sexual IPV: 23/87 = 26.7% Among those who do not report sexual IPV: 29/162 = 17.9% Prevalence of problem drinking: Among those who report physical IPV: 37/142 = 25.9% Among those who do not report physical IPV: 15/107 = 14.2% Prevalence of problem drinking: Among those who report psychological IPV: 47/204 = 23.2% Among those who do not report psychological IPV: 5/45 = 11.1% Prevalence of problem drinking: Among those who report any IPV: 49/214 = 23% Among those who do not report any IPV: 3/35 = 8.6%	Sexual IPV (with or without psychological or physical IPV): aOR 3.84; 95% CI 1.04, 14.26 Physical IPV with/without psychological IPV: aOR 3.52, 95% CI 0.92–13.49 NS Psychological IPV: aOR 2.02, 95% CI 0.50–8.24 NS No statistical measure of association conducted for Any IPV	Total score: 25/42 Selection score: 4/14 Measurement score: 12/14
Foran, 2014	42,744 active duty US Air Force members (34,713 males; 8031 females) from 82 bases worldwide in current relationship. All active duty members (*N* = 128,950) of United States Air Force invited to participate	Past-year clinically significant emotional abuse (CS-EA)^f^	Past-year prevalence CS-EA (weighted): males 6%; females 8.5%	Alcohol Use Disorders Identification Test (AUDIT) (measures symptoms over past year, with higher scores indicating more alcohol problems)	No data provided	Males: aOR 1.34, 95% CI 1.26–1.33; Females: aOR 1.34, 95% CI 1.17–1.53	Total score: 40/42 Selection score: 14/14 Measurement score: 14/14
Forgey, 2010	Study invitation letter mailed to every enlisted female married to civilian spouse at large Army installation (*n* = 248)	Any past-year (physical/sexual/psychological) IPV measured using Conflict Tactics Scale-2 (CTS-2)	Past-year unilateral IPV prevalence, male perpetrator: 42/248 (16.9%)	Michigan Alcoholism Screening Test (SMAST), cutoff ≥2 (measures symptoms over past year)	No statistically significant difference in females’ drinking behaviour found—no data provided	No measure of association conducted in study	Total score: 22/42 Selection score: 6/14 Measurement score: 5/14
Iverson, 2015	160 randomly selected New England female VA patients with intimate relationship in past year	Any past-year IPV (physical or psychological) measured using Hurt, Insult, Threaten, Scream (HITS) tool	Past-year IPV prevalence: 58/160 (36.3%)	AUDIT, cutoff = 8 (measures symptoms over past year) Prevalence of probable alcohol dependence^g^: 16/155 (10.3%)	Prevalence of probable alcohol dependence: Among those who report IPV: 10/58 = 17.2% Among those who do not report IPV: 6/102 = 5.9%	aOR 2.88 (95% CI 0.94–8.82) *p* = 0.06 NS	Total score: 31/42 Selection score: 5/14 Measurement score: 13/14
Rosen, 2002	488 (358 White males; 130 Black, males) married soldiers at Army post in Alaska. On average, approximately 60% of personnel from available units provided with opportunity to complete questionnaire	Any past-year physical, and any past-year psychological IPV measured using modified CTS (MCTS)	Prevalence of minor physical IPV: 114/488 (23.4%) Severe physical IPV: 78/488 (16%) Prevalence rate for psychological IPV not reported	SMAST measures symptoms over past year Mean alcohol score (range 0–1): 0.50	Data not provided	MANCOVA: Severe physical IPV: *f* = 4.8, *p* < 0.05 Mild physical IPV: *f* = 0.1 NS Psychological IPV: *f* = 5.4, *p* < 0.05	Total score: 25/42 Selection score: 6/14 Measurement score: 9/14
Zamorski, 2013	1745 randomly selected (1017 males; 728 females) Regular active duty Canadian Armed Forces members in current relationship	Any IPV (physical/sexual/psychological—including financial abuse) experienced over the course of the current relationship measured using Canadian General Social Survey	(Weighted %) Any physical and/or sexual IPV: both males and females 170/1745, 15.4%^d^ (95% CI 12.3–18.5) Any emotional and/or financial IPV: both males and females 387/1745, 25.1%^d^ (95% CI 21.6–28.7)	AUDIT, cutoff ≥8 men, ≥7 women (measures symptoms over past year) (Weighted %) Prevalence of hazardous and harmful drinking: 248^a^/1551 = 18% (95% CI 14.7–21.2)	(Weighted %) Prevalence of hazardous and harmful drinking: Among those who report physical and/or sexual IPV: 39/241^a^ = 22.3% (95% CI 13.4–31.3) Among those who do not report physical and/or sexual IPV: 111/1275 = 14.9% (95% CI 11.3–18.5) (Weighted %) Prevalence of hazardous and harmful drinking: Among those who report emotional and/or financial IPV: 85/240 = 41% (95% CI 31.0–51.1) Among those who do not report emotional and/or financial IPV: 258/1273 = 22.7% (95% CI 18.7–26.7)	Physical and/or sexual IPV: aOR 1.50, 95% CI 0.77–2.92 NS Emotional and/or financial IPV: aOR 2.01, 95% CI 1.17–3.43	Total score: 37/42 Selection score: 11/14 Measurement score: 13/14
Mental health multi-morbidity							
Iverson, 2015	160 randomly selected New England female VA patients with intimate relationship in past year	Any past-year IPV (physical or psychological) measured using Hurt, Insult, Threaten, Scream (HITS) tool	Past-year IPV prevalence: 58/160 (36.3%)	Prevalence of mental health multi-morbidity (2 or more of the following: PTSD, depression and alcohol misuse): 50/156 (32.1%)	Prevalence of Morbidity: Among those who report IPV: 29/58 = 50% Among those who do not report IPV: 21/102 = 20.6%	aOR 3.32, 95% CI 1.54–7.17	Total score: 31/42 Selection score: 5/14 Measurement score: 13/14

^a^Total *N* varies due to missing data

^b^Group 6: Male Civilian Spouse perpetrator—included all cases in which the enlisted female reported that her spouse alone had been violent towards her or that her spouse had committed a more severe form of violence towards her than she had committed towards him

^c^Weighted data

^d^Weighted  %

^e^Total *N* varied by outcome

^f^Operationalised as (a) at least one reported emotionally aggressive act that caused (b) significant stress, depression, or fear (for the victim’s own safety or that of someone she cared about) that interfered with their functioning. Internal consistency of ten emotional abuse acts comparable with CTS-2

^g^A cut-off score of 8 was used to develop a dichotomous probable alcohol dependence variable

Studies examined IPV measured over a variety of time periods. Four studies reported on past-year IPV [50, 54, 84, 85], six studies on lifetime [47, 48, 58, 81–83], two studies on IPV experienced over the course of the current relationship [44, 45], and one study on IPV experienced during military service [52]. Further details of sample size, study methods, and findings are presented in Tables 2 and 3.

### Main findings

#### Depression

Nine studies examined depressive symptoms among individuals who have experienced IPV victimisation [44, 45, 47, 48, 52, 54, 58, 84, 85], with four studies rated as high quality [44, 45, 47, 48]. Six studies found a statistically significant association between IPV victimisation and depression after taking account of potential confounders [44, 45, 47, 48, 54, 84], of which four were rated as high quality.

The majority of study findings will be reported according to gender. However, two high-quality studies explored the association between depression and IPV experienced over the course of the current relationship among samples of male and female Canadian Armed Forces (CAF) members and did not stratify analyses by gender. The first study used a representative sample of 1745 CAF members and found that ‘probable depression’ was significantly associated with increased emotional and/or financial abuse, but not with any physical and/or sexual IPV [44]. The second study (*n* = 529) similarly found that emotional violence victimisation (defined as experiencing threats of violence) was significantly associated with depression [45], but physical violence victimisation was not.

##### Female victims

In a study of past-year IPV among active duty females married to civilian spouses (*n* = 248), the researchers grouped participants according to six different patterns of violence (depending on gender of perpetrator and unidirectional/bi-directional violence of differing severities). It was found that mean depression scores were significantly higher among females who reported experiencing violence by a male civilian spouse compared to those who reported no violence. Mean depression scores did not differ significantly between the no violence group and the group in which the more severe violence was perpetrated by the enlisted female. The mean depression score for the group in which both the enlisted female and her spouse had engaged in severe violence and/or injury was significantly higher than all other group scores [85]. This study did not conduct a statistical analysis of the association between depression and IPV. Another study which utilised a clinical sample of female Veterans Affairs (VA) patients found that of those who reported any type of IPV (defined as physical and/or psychological) victimisation in the past year 67.2% were categorised as cases of depression, compared to 18.6% of those who did not report IPV. On further analysis, any past-year IPV was significantly associated with depression [84].

With regard to lifetime IPV, a high-quality study utilised data from a nation-wide telephone survey of non-institutionalised adults in the US and found that among female veterans (503 out of a total *n* = 21,162 females) IPV was significantly associated with increased cases of depression [47] (see also Table 3). By comparison, a study of active duty females (*n* = 616) found no significant association between physical and/or sexual IPV and cases of depression [58]. Finally, a study exploring sexual IPV experienced by female VA patients (*n* = 369) during military service found no significant difference between the mean depression score of women who experienced sexual IPV compared to those who had not experienced sexual abuse [52].

##### Male victims

One study of male active duty Army personnel (*n* = 488) found that past-year physical and psychological aggression was significantly associated with depression [54]. The researchers split their sample according to ethnicity and found that severe physical IPV victimisation was more strongly associated with depression for Black than for White soldiers. Similarly, a high-quality community-based study (4356 male veterans out of a total *n* = 13,765 males) found that among veterans lifetime IPV (any IPV defined as actual or threatened physical violence or unwanted sex) was significantly associated with increased depression [48].

#### Post-traumatic stress disorder (PTSD)

Five studies analysed the association between IPV victimisation and PTSD [44, 52, 58, 83, 84], with one study rated as high quality [44]. Three studies (none were high quality) found a significant association between IPV and PTSD after taking account of potential confounders [52, 83, 84]. Again, study results will mostly be reported by gender, but one high-quality study of CAF members which utilised a mixed gender sample did not stratify analyses by gender. This study found no significant association between any physical and/or sexual IPV or any emotional and/or financial IPV experienced over the course of the current relationship and PTSD [44].

##### Female victims

A study of 160 female VA patients found that 41.4% of females who reported past-year IPV had a PTSD diagnosis, compared to 19.6% of those who did not report IPV, and past-year physical and/or psychological IPV was found to be significantly associated with PTSD [84]. Similarly, a study of female VA patients (*n* = 1206) found that 32.2% of those who reported lifetime IPV had a PTSD diagnosis, compared to 14.8% of those who did not report IPV, and PTSD was significantly associated with physical IPV [83]. In contrast, a military population-based study of active duty tri-service females (*n* = 616) did not find a significant association between physical and/or sexual lifetime IPV and PTSD [58]. Finally, one study found that women who experienced sexual IPV during military service had significantly higher levels of PTSD symptoms compared to women without a history of sexual IPV [52]. The PTSD scores of women who experienced sexual IPV by an intimate partner were not significantly different to those of women who experienced sexual abuse perpetrated by a non-intimate partner.

#### Alcohol/substance use problems

Seven studies explored alcohol misuse among individuals who have experienced IPV [44, 50, 54, 81, 82, 84, 85], with two studies rated as high quality [44, 50]. Four studies found a statistically significant association after taking account of potential confounders [44, 50, 54, 82] with two of these being high quality. One high-quality study with a mixed gender active duty sample did not stratify analyses by gender. It was found that experience of any emotional and/or financial abuse victimisation over the course of the current relationship was associated with high-risk drinking [44]. No significant association was found between physical and/or sexual IPV and high-risk drinking.

##### Female victims

A high-quality study utilising a representative sample of active duty US Air Force members (*n* = 42,744; 8031 females) found that past-year clinically significant emotional abuse (defined as at least one reported act that caused significant distress that interfered with the victim’s functioning) was significantly associated with alcohol problems [50]. In contrast, two smaller and lower quality studies did not find different drinking patterns among those reporting past-year IPV compared to those without IPV [85] and did not find an association between IPV and probable alcohol dependence [84].

Two studies examined lifetime IPV victimisation and alcohol problems among clinical populations of female VA patients [81, 82]. One study (*n* = 249) found a significant association between sexual IPV (with/without physical or psychological IPV) and problem drinking, but no association with either physical (with/without psychological IPV) or psychological IPV [82]. The other study (*n* = 2670) reported that lifetime physical IPV increased significantly with AUDIT-C scores of five or more [81]. However, no statistical analysis of the association between IPV and alcohol problems was conducted (see also Table 3).

##### Male victims

A high-quality study of 34,713 male US Air Force members found that clinically significant emotional abuse was significantly associated with alcohol problems [50]. Another study of active duty males found that severe physical past-year IPV and psychological IPV were associated with alcohol problems [54]. No significant association was found between mild physical IPV and alcohol problems.

#### Mental health multi-morbidity

A study of 160 female VA patients found that of those who reported IPV, 50% reported mental health multi-morbidity (defined as the presence of at least two of the following conditions: depression, PTSD, alcohol dependence), compared to 20.6% of those who did not report IPV. Past-year physical and/or psychological IPV victimisation was significantly associated with mental health multi-morbidity [84].

## Discussion

### Summary of main findings

The aim of this review was to explore the association between IPV victimisation and mental health problems among current and former military personnel. The number and quality of studies which found an association between IPV and depression/alcohol problems was higher than for IPV and PTSD. An association between IPV and mental health problems was more frequently found in studies of veterans compared to active duty personnel. However, the link between IPV and alcohol misuse was more consistently found among active duty samples. Among active duty personnel, psychological IPV was more consistently associated with depression/alcohol problems than physical/sexual IPV.

### Link between IPV and mental disorder among military personnel

Six of the seven studies that examined the association between IPV and depression found a significant association after controlling for potential confounders, with four studies being of high quality. All studies that explored psychological IPV and depression found a significant association [44, 45, 54]. Evidence for an association between physical and/or sexual IPV and depression was less consistent, with three out of four studies finding no significant association (two of which were high quality). Veteran studies tended not to look at IPV sub-types, but consistently found associations between IPV and depression. Of note, the two high-quality studies that reported on both males and females together found that depression was associated with psychological IPV, but not with physical/sexual IPV [44, 45]. Overall, the number and quality of studies finding an association between IPV victimisation and depression were similar between males and females. A bi-directional causal relationship has been found between IPV and depression in the general population, though there are limited data on this relationship for men [86, 87]. A traumatic stress response framework has frequently been used to conceptualize the link between IPV and depression: traumatic events such as domestic abuse can cause fear, stress, and feelings of helplessness, isolation, and powerlessness, which may lead to depression [86, 88–92]. Salcioglu et al. [93] found that the strongest predictors of depression and PTSD in IPV survivors were helplessness and fear due to a sense of ongoing threat to safety. It has been suggested that a chronic traumatic stress response, where a victim is subjected to ongoing abuse, may lead to alterations in affect and sense of self (i.e., the predominance of self-blame and depressive affect) [94–96]. Common risk factors exist between IPV and depression, such as demographics, childhood adverse events, and substance use, which would need to be controlled for in research into this association [86].

Three of the five studies that examined the association between IPV and PTSD found a significant association after controlling for confounders. All three studies utilised female veteran samples, and none were rated as high quality. Two studies of active duty personnel (one was high quality) did not find a statistically significant association after adjustment for confounders [44, 58] (see Table 3). Only one study included male participants, but the sample was mixed gender and analyses were not stratified by gender. Therefore, it was not possible to comment on differences between males and females. There were too few studies to comment on differences in the association between sub-types of IPV and PTSD.

Four of the five studies (two were high quality) that investigated the association between IPV and alcohol problems found a significant association after controlling for potential confounders. An association between IPV and alcohol problems was more consistently found among male compared to female personnel in this review. However, it should be noted that the one high-quality study providing separate data on males and females reported identical odds ratios for emotional IPV and alcohol problems [50]. Among males and in active duty samples, psychological IPV was consistently found to be associated with alcohol problems [44, 50, 54]. Among females, the results were mixed. Only one veteran study explored the association between psychological IPV and alcohol problems, and no significant association was found. Overall, psychological IPV was more consistently found to be associated with alcohol problems than physical IPV.

The National Violence Against Women Survey (NVAWS) of males and females aged 18–65 found that psychological IPV was more strongly associated with adverse health outcomes (including depressive symptoms and substance use) than physical IPV [65]. The findings of the current review also support this: among active duty personnel, psychological IPV was more consistently associated with depression/alcohol problems than physical/sexual IPV. Previous research has found that perpetrators of IPV are more likely to disclose psychological than physical abuse [97]. It is possible that a similar pattern is present among victims of IPV, though for perhaps different reasons. Wider research has found that barriers to the disclosure of IPV among mental health service users include fear of the consequences (including fear of Social Services involvement/child protection issues) and feelings of shame [98]. It is possible that victims perceive these barriers to disclosure to be greater in the context of physical/sexual than psychological violence.

### Veteran vs active duty

Significant associations between IPV and depression/PTSD were more consistently found among veterans than active duty personnel. Research has confirmed the under-reporting of mental health problems among serving military personnel [99, 100]. Identified barriers to help-seeking include feared impact on an individual’s military career [101–106] and also practical barriers such as lack of time due to a busy schedule [100, 105, 107]. A recent meta-analysis described the most frequently reported deterrents to seeking help for mental health problems; “My unit leadership might treat me differently” and “I would be seen as weak” [108]. Service providers working with military couples have observed that these barriers mean that personnel may be more open to seeking help after their military career has ended [109]. Furthermore, it has been found that the wives of active duty males refrained from disclosing IPV victimisation as they wanted to appear strong and capable to avoid being perceived as a “failed” military wife [110]. There is no similar research among samples of serving personnel who are victims of IPV, but such barriers to disclosure of abuse may exist.

Research is also emerging showing higher rates of mental health and social problems among veterans than among active duty personnel [41, 111]. A number of factors may contribute to this, such as the impact of transition [37, 112], loss of role or identity [30], and fragmentation of the social support network enjoyed in the military [113]. These factors could lead to the apparent increased association between IPV and mental health problems among veterans compared to active duty personnel.

The number and quality of studies finding an association between IPV and alcohol problems were found to be higher among active duty than veteran samples. There is a culture of excess alcohol consumption in the military [114]. It has been observed that military culture “fosters a warrior ethos that rewards physical and emotional prowess and frowns upon weakness and timidity” [115]. It is possible that active duty personnel perceive depression/PTSD to be more closely associated with weakness and, therefore, less acceptable than alcohol problems, thereby leading to the under-reporting of the latter. It is also possible that use of alcohol is a coping mechanism that masks the symptoms of other mental disorders. Alcohol misuse is highly comorbid with mental disorders such as depression and PTSD among military personnel [116].

### Impact of gender

Perhaps, the most striking finding of this review was the lack of research into male IPV victimisation and mental health. Research in the general population has shown that women are at greater risk of IPV victimisation compared to men, and the psychiatric burden of IPV is greater among women [117]. Research into IPV and mental health among women in the military is, therefore, necessary. However, the National Violence Against Women Survey (NVAWS) found that, for both men and women, IPV victimisation was associated with increased risk of current poor health, depressive symptoms, substance use, chronic mental illness, chronic physical disease, and injury [65]. This is supported by further research in the general population that IPV can impact significantly on the psychological health of male victims [65, 118, 119]. The military culture, that favours male strength and is forbidding of male weakness, may have influenced the direction of research to focus on female IPV victims. However, it has been noted that to frame the problem as ‘violence against women’ overlooks males who may be victims of violence in gender-saturated contexts, such as IPV [120]. Walby et al. [121] argue that if the focus in official crime statistics is biased towards women, then we cannot explore the gendered nature of violence, which requires comparisons between males and females.

Studies in both the general population and military samples have found that men and women are equally likely to be violent in intimate relationships, but women are more likely to suffer an injury and are at greater risk of serious and sexual assaults [120, 122–128]. Not only were there too few studies of male victimisation to compare the impact of IPV on mental health by gender, but studies also neglected to measure impact of IPV. The only study that considered impact [85] found that in almost two-thirds of the cases of bi-directional violence of differing levels of severity (15.5% of all violence), the more severe violence was perpetrated by the male civilian spouse. A higher prevalence of injury was found among females (16.4%) compared to males (11%) [85]. However, it is important to note that in this study, enlisted females were asked to report on both their own and their spouse’s behaviour, and therefore, there is likely to be significant reporting bias. Considering that, in the general population, the proportion of homicides committed by an intimate partner is six times higher for female (38.6%) than for male (6.3%) homicides [126], it seems that gender differences in IPV victimisation in this review may be masked by the lack of measurement of the impact of IPV. Walby et al. [121] assert that the gendered lack of alignment between actions and impact/consequences means that actions alone cannot be relied upon to define a violent event. Consequently, the authors argue that the CTS [129] is not an appropriate tool to measure violence, as it focuses on actions only and excludes impact/consequences, meaning that it is incompatible with the concept of crime used in criminal justice systems.

### Strengths and limitations

To our knowledge, this is the first systematic review of studies of IPV victimisation and mental disorder among military populations. The strengths of this review are that it included studies of psychological and sexual IPV, rather than just physical violence, and it only included studies that used validated tools to measure symptoms of mental disorder. The interpretation of the review findings was limited by heterogeneity among the included studies. Diverse tools were used across studies to measure IPV and there were variations in the timing of IPV studied (for example, past-year or lifetime, IPV experienced over the course of the current relationship, or during military service). These inconsistencies made comparisons between studies difficult.

Problems with IPV measurement were not only a significant limitation of all studies in this review, but also are a criticism of the field of IPV research as a whole [130–132]. IPV research has been based on varied and poorly-defined definitions of the types of IPV, particularly of “Any” IPV, hindering meaningful comparisons between studies [130]. Research findings are potentially distorted by reliance on participants’ self-report on their partners’ behaviour [130, 133, 134]. There is also no consensus as to whether threats of physical harm should be measured by physical abuse scales, or psychological abuse tools [135]. This is problematic considering that, not surprisingly, methods of IPV measurement have a powerful influence on study findings [131].

Only two studies in this review considered differences in the severity of IPV [54, 85]. It has been observed that combining individuals, who experience a high frequency of mild incidents with those experiencing a low frequency of severe violence, may result in distortions when making comparisons across research [130, 136]. It has been observed that IPV is often reciprocal and frequently occurs during interpersonal events. However, there is little acknowledgement of this in current methods of IPV measurement [137]. Only one study in this review considered whether victims also perpetrated violence, and found that over 60% of all reported violence was bi-directional [85]. The lack of consideration of patterns of violence between couples may have led to some misclassification bias among studies. IPV measures have been criticised for a lack of consideration of the context of abusive actions, for example, not excluding physically forceful acts that are used in self-defence [138]. However, there is no consensus on the specific contexts (e.g., retaliation) that should be examined to ensure accuracy of data collection [131]. Follingstad and colleagues suggest that continuing with the current approach to measuring IPV hinders the improvement of the current evidence base, and stresses the importance of developing a “gold standard” measurement that would allow for meaningful comparison of research findings [131].

All studies included in this review used validated tools to measure mental disorder. However, some measured symptoms rather than providing a diagnosis, limiting the reliability and comparability of study findings. Studies did not consistently control for potential confounders when examining the association between IPV and mental disorder. Finally, all included studies were cross sectional, meaning that no conclusions can be drawn regarding the direction of causality between IPV victimisation and mental disorders.

### Implications

The findings from this review indicate that, just like among civilian populations [65], the burden of mental health need may be significant among military personnel who are victims of IPV. This emphasises the important role of health as well as welfare workers in the identification and management of IPV and its consequences. We need research to help us better understand barriers to the reporting of IPV in military culture, in order that effective interventions can be developed.

IPV is associated with adverse health consequences for both male and female victims [65], and there is considerable evidence that men are less likely than women to seek help for diverse mental and physical health problems [139]. In the UK, Joint Service Publication (JSP) policies detailing procedures for military welfare provision surrounding IPV have been developed based on the Ministry of Defence’s commitment to support the cross government Violence Against Women and Girls agenda [140]. Notably, although the JSP policy acknowledges the possibility of male victimisation in its definition of IPV, the sections providing practice direction for IPV cases and detailing safety planning procedures focus on the victim being female [141]. This is suggestive of a lack of focus on male victimisation, which may be exacerbated by persistent attitudes towards gender roles in military culture and is supported by the lack of research on males in this review. IPV awareness and management is more advanced in the US military, most likely driven by the larger body of research literature (all studies in this review were based in the USA or Canada), where there is greater emphasis on prevention strategies [142] and they differentiate between civilian and serving victims [143]. In the US, victim advocate services and the Family Advocacy Program are widespread [143]. Domestic violence advocacy has been introduced in the UK in recent years [66, 70]; however, research trialling these methods in military environments needs to be conducted, as it has been in the US [144].

This review highlights the need for further research to examine IPV victimisation and mental disorder among active duty and veteran military personnel. There is a need for greater consistency in IPV measurement to allow meta-analyses of the findings of different studies. Future research should consider the impact of IPV victimisation in order that gender differences can be better understood.

## Electronic supplementary material

Below is the link to the electronic supplementary material.

Supplementary material 1 (DOCX 43 kb)

## Footnotes

1 We use ‘military couple’ to mean a couple in which one or both the partners is serving or has served in the military.
