# Supplementary material for: Systematic review of mental health disorders and intimate partner violence victimisation among military populations
Source: Soc Psychiatry Psychiatr Epidemiol. 2017 Jul 26;52(9):1059–80. doi: 10.1007/s00127-017-1423-8 (PMC5581819; doi:10.1007/s00127-017-1423-8)
Supplement: Supplementary file 1 — Supplementary material 1 (DOCX 43 kb) [file 127_2017_1423_MOESM1_ESM.docx]

**Literature review search strategy and search terms**

Literature review title:

Systematic review of mental health disorders and intimate partner violence victimisation among military populations.

1. **Objectives**

The objective of this review is to:

1. Identify mental health problems associated with IPV victimisation among male and female military personnel (both serving and ex-serving).
2. **Key terms**
3. **Intimate partner violence (IPV)**

This review adopts the UK Home Office definition of IPV and includes violence perpetrated against female and male partners:

*Any incident of threatening behaviour, violence or abuse (psychological, physical, sexual, financial or emotional) between adults who are or have been intimate partners or family members, regardless of gender or sexuality [*[*1*](#_ENREF_1)*].*

1. **Mental health problems**

Search terms for mental health problems were adapted from NICE guidelines [[2](#_ENREF_2)].

1. **Selection criteria**
   1. **Inclusion criteria**

**Study Population**

Studies will be eligible for inclusion in the review if samples include regular or reserve military personnel and veterans and their intimate partners.

**Study Characteristics**

Quantitative studies are eligible for inclusion if they present the results of peer-reviewed research. All study designs are eligible for inclusion if they allow calculation of prevalence of IPV.

- 1. **Date Restrictions**

Databases were searched from their dates of inception up to 31st May 2016.

- 1. **Exclusion criteria**
- Studies that focus on military personnel as perpetrators rather than victims of violence.
- Studies that focus on military sexual trauma, or veterans’ experience of sexual violence or harassment within the military perpetrated by someone other than an intimate partner.
- Studies that focus on interpersonal violence in the military, but not in the home.
- Studies that utilised qualitative methods or a quantitative study from which a prevalence statistic could not be extracted.
- Studies with sample sizes of <100 participants.
  1. **Language Restrictions**

Studies will only be eligible for inclusion in the review if they are published in English.

1. **Data Sources**

The review will search multiple electronic databases and relevant websites. Reference lists of all included studies will also be searched and forward citation tracking used to identify additional potentially relevant studies. Reference lists of key literature reviews, which examine the objectives stated in section 1, will also be searched to identify potentially relevant studies.

1. **Electronic Databases**

The following biomedical and social sciences databases will be searched:

- Embase
- Medline
- psycINFO
- Web of Science

1. **Contacting Key Experts**

The reviewers will contact some of the corresponding authors of included papers, asking them for further information about study methodology and outcomes (if required).

1. **Conducting the Review*:***
   1. **Study appraisal**

Study quality will be independently appraised by two reviewers using criteria adapted from previously validated tools.  Criteria relate, for example, to the study design, the representativeness of study samples, the measurement of study outcomes, and the appropriate use of statistical analysis (see Appendix C).  Adaptations to a critical tool developed by Loney et al ([Loney, Chambers et al. 2000](file:///L:\PROVIDE%20Study\Protocol\Systematic%20Review%20Protocol_Quant_Perp.12.01.10.docx#_ENREF_16)), incorporating a number of questions on study methodology from the Critical Appraisal Skills Program (CASP) checklists [[3](#_ENREF_3)], confounding and attrition from Downs and Black ([Downs and Black 1998](file:///L:\PROVIDE%20Study\Protocol\Systematic%20Review%20Protocol_Quant_Perp.12.01.10.docx#_ENREF_6)), and the quality of assessment of mental disorder from Saha et al ([Saha, Chant et al. 2005](file:///L:\PROVIDE%20Study\Protocol\Systematic%20Review%20Protocol_Quant_Perp.12.01.10.docx#_ENREF_20)).

The quality appraisal form has 21 questions about study quality. Papers receive a grade of between 0 and 2 for each question, giving a maximum total score of 42. A study is awarded 0 points if it does not meet the criteria or answer the question, 1 point if it partially meets the criteria or gives a partially satisfactory answer to the question, and 2 points if it fully meets the criteria or gives a fully satisfactory answer to the question. Overall study quality and scores on domains relating to selection and measurement bias will be assessed according to the percentage of the maximum possible quality score attained and categorised into high or low quality on the basis of their score in order to determine eligibility for inclusion in the systematic review.

- 1. **Title and Abstract Screening**

Literature searches of the databases listed in section 5 will be conducted and the resulting citations will be downloaded to EndNote© software, where duplicate citations will be removed. Additional citations that have been identified by forward and backward citation tracking, hand searches, key experts and the re-examination and updating of systematic reviews will also be added to the EndNote© database.

Based on the criteria described in section 3 the titles and abstracts of all downloaded citations will be evaluated for a decision on initial inclusion or exclusion^[[1]](#footnote-1)^. All studies and documents identified during the database search will be assessed for relevance to the review based on the information provided in the title, abstract, and descriptor/MeSH terms. Studies identified from reference list searches will be assessed for relevance to the review questions based on the study title, abstract and descriptor/MeSH terms. Hard copies of the papers identified at this stage as potentially eligible for inclusion will be obtained. Excluded citations will be retained in separate folders within EndNote©, categorised according to the primary reason for exclusion. If it is unclear whether a paper meets the inclusion criteria based on the title and abstract, it will be taken forward to the next stage of screening.

- 1. **Retrieval and Review of Full Text Articles**

In the case of not having access to papers the authors will be contacted directly to ask for access, if this is not granted the paper will be omitted from the review.

References:

1. Home Office (2005) Domestic violence: a national report. . Home Office, London

2. NICE (2011) Common mental health problems: identification and pathways to care. Clinical guideline [CG123]<https://www.nice.org.uk/guidance/cg123>

3. CASP UK (2017) Critical Appraisal Skills Programme (CASP): Making sense of evidence. <http://www.casp-uk.net/casp-tools-checklists>

**Appendix A**

Author Name:

|  |
| --- |

Paper title:

|  |
| --- |

Reviewer ID:

|  |
| --- |

**CHECKLIST**

Does the paper meet **each** of the following inclusion criteria?

| **Inclusion criteria** | **If yes tick box** |
| --- | --- |
| Study is published in a peer-reviewed journal |  |
| Study uses an eligible study design (randomised controlled trial, non-randomised controlled trial, before and after study, interrupted time series study, parallel group study, cohort study, case-control study, cross-sectional study) |  |
| Sample includes military participants or veterans and/or their intimate partners |  |
| Study results include prevalence, risk or odds of psychological adjustment measures intimate partner violence perpetration/victimisation (section 2), or presents data from which these statistics can be calculated |  |

Does the paper meet **any** of the following exclusion criteria?

| **Exclusion criteria** | **If yes tick box** |
| --- | --- |
| Study is published in a book, thesis/dissertation, conference paper, general comment paper, letter, editorial, report, or other non-peer reviewed format. |  |
| Study uses an ineligible study design (single case study, case series analysis, qualitative interview, focus group interviews) |  |
| Study does not measure victimisation of intimate partner violence. |  |
| Study results do not include prevalence, risk or odds of domestic violence perpetration, and does not present data from which these statistics can be calculated. |  |

**Is the paper eligible for inclusion (Y/N):**

If the paper meets any of the exclusion criteria do not proceed with data extraction.

**Appendix B**

**Search terms**

Search terms were derived from looking at MeSH terms in PubMed, using sources such as the Personnel Recovery Unit (PRU) recovery route leaflet and the Army Recovery Capability website.

Abuse terms used:

- Intimate partner violence
- Intimate partner violence victimisation
- Domestic violence
- Domestic violence victimisation
- Violence
- Domestic abuse
- Domestic abuse victimisation
- Abuse
- Family violence
- Intimate partner abuse
- Spousal abuse
- Partner abuse

Military terms used:

- Military
- Army
- Armed Forces
- Military personnel
- Military service personnel
- Air Force*
- Marine
- Navy
- Soldier
- Veteran*

Victimisation terms used:

- Victim
- Victimisation
- Victim*
- Victimi*ation

Mental health terms

- (Mental health problem* OR difficult* OR disorder* OR ill*)
- Depression
- Mood disorder
- PTSD
- Post-traumatic stress disorder
- Anxiety
- Stress
- Psychosis
- Bipolar
- Mania OR manic
- Obsessive OR compulsive
- Eating disorder*
- Adjustment disorder
- Neurotic

**Combinations used**

1. domestic violence victimisation (exp domestic violence, abuse, victim, family violence) AND military (exp army)
2. domestic violence AND victim AND military
3. intimate partner violence AND victim AND military
4. military AND violence AND victim
5. domestic abuse (exp domestic violence, partner violence, family violence, abuse) AND Armed Forces (exp army)
6. (domestic abuse OR domestic violence) AND (military personnel OR military service personnel) AND victim*
7. (domestic abuse OR domestic violence) AND victim*

*AND*

Armed Forces OR Air Force OR Marine* OR Navy OR soldier

1. domestic abuse victimisation AND (military OR Armed Forces OR Air Force* OR Marine* OR Navy OR soldier)
2. domestic abuse AND victim AND (military OR Armed Forces OR Air Force* OR Marine* OR Navy OR soldier)
3. (military OR Armed Forces OR Air Force* OR Marine* OR Navy OR soldier) AND abuse AND victim
4. (Intimate partner abuse OR spousal abuse) AND (military personnel OR military service personnel)
5. (partner abuse OR spousal abuse) AND veteran*
6. (domestic abuse OR domestic violence) AND veteran*
7. Intimate partner violence AND victim AND (veteran* OR military personnel OR military service personnel OR soldier)
8. (intimate partner abuse OR domestic abuse) AND victim AND (veteran* OR military personnel OR military service personnel OR soldier)
9. (intimate partner violence OR domestic abuse OR domestic violence) AND victim AND (veteran* OR military personnel OR military service personnel OR soldier)
10. (intimate partner violence OR domestic abuse OR domestic violence) AND victim AND (military OR Armed Forces OR army)
11. 1-17

*AND*

(mental disorder* OR mental ill* OR mental difficult* OR mental health problem*) OR depression OR mood disorder OR PTSD OR post-traumatic stress disorder OR anxiety OR stress OR psychosis OR bipolar OR mania OR manic OR obsessive OR compulsive OR eating disorder* OR adjustment disorder OR neurotic

1. Victim* AND (abuse or violence)

*AND*

(military OR Army OR Armed Forces OR Air Force OR Marine* OR Navy OR soldier OR military personnel OR military service personnel)

*AND*

(mental disorder* OR mental ill* OR mental difficult* OR mental health problem*) OR depression OR mood disorder OR PTSD OR post-traumatic stress disorder OR anxiety OR stress OR psychosis OR bipolar OR mania OR manic OR obsessive OR compulsive OR eating disorder* OR adjustment disorder OR neurotic

1. (intimate partner violence OR domestic abuse OR domestic violence) AND victim*

*AND*

(military OR Army OR Armed Forces OR Air Force OR Marine* OR Navy OR soldier OR military personnel OR military service personnel)

*AND*

(mental disorder* OR mental ill* OR mental difficult* OR mental health problem*) OR depression OR mood disorder OR PTSD OR post-traumatic stress disorder OR anxiety OR stress OR psychosis OR bipolar OR mania OR manic OR obsessive OR compulsive OR eating disorder* OR adjustment disorder OR neurotic

**Appendix C**

**Quality Appraisal Form**

**Please complete part 1 for all study designs and complete the relevant sections for part 2, specific to study design.**

Score the answer to each question by ticking 0, 1 or 2:

0 – study does not meet criteria/answer question

1 – Study partially meets criteria/gives a partially satisfactory answer to the question

2 – Study fully meets criteria/gives a fully satisfactory answer to the question

| **Part 1** | | | | | | | | | |
| --- | --- | --- | --- | --- | --- | --- | --- | --- | --- |
| **Screening questions** | | | | | | **Score** | | | |
|  | Question | | Comments | | | 0 | | 1 | 2 |
| 1 | Did the study ask a clearly focused question?  *– Is the hypothesis/aim/objective of the study clearly described?*  *-Is the study question focused in terms of the outcomes considered?* | |  | | |  | |  |  |
| 2 | Is the study design appropriate for the research question? | |  | | |  | |  |  |
| 3 | Was a validated tool used to assess mental disorder?   - - *Diagnostic interview using validated instrument, e.g., SCID =2*   - *Screening instrument for mental disorder e.g. PHQ, GAD-7, CES-D* | |  | | |  | |  |  |
| **Continue only if score on each of questions 1 and 2 is one or more** | | | | | | | | | |
| **Detailed questions** | | | | | | | | | |
| **Measurement of risk of selection bias** | | | | | | | | | |
| 4a | | Is the sampling method appropriate for the research question?  *Consider:*  *-The sampling method used (i.e. random selection of subjects)*  *- If applicable, is there appropriate selection of controls?* | |  |  | |  | |  |
| 4b | | Are subjects appropriately defined?  *Consider:*  *- Inclusion/ exclusion criteria specified*  *- Inclusion/exclusion criteria appropriate* | |  |  | |  | |  |
| 4c | | Is the sample size appropriate?  *Consider:*  *- Is the sample size justified?*  *- Were a sufficient number of cases selected?*  *- If applicable, were a sufficient number of controls selected?* | |  |  | |  | |  |
| 4d | | Is the study sample representative of the population of interest?  *-Do the authors assess the representativeness of the study sample?* | |  |  | |  | |  |
| 4e | | Does the level of non-participation risk introducing bias?  *Consider:*  *-Are key demographic characteristics of non-participants reported and compared against participants?*  *-Does the study report on the impact of non-participation?*  *-If applicable, rates of attrition reported* | |  |  | |  | |  |
| 5 | | Is the study setting appropriate to the aims of the research? (e.g. setting, location, relevant dates) | |  |  | |  | |  |
| 6 | | Is the method of data collection appropriate for the aims of the research? | |  |  | |  | |  |
| **Measurement of risk of measurement bias** | | | | | | | | | |
| 7 | | Are suitable/standard criteria used for measurement of domestic violence?  *Consider:*  *-Criteria of domestic violence was clearly defined*  *-Potential for bias of measurement*  *-If measures piloted*  *- Standardised/pre-validated measures (score 2 points)*  *- Researchers developed their own measure (score 1 point)*  *- No details of measurement were provided (score 0 point)* | |  |  | |  | |  |
| 8 | | Are known confounders accounted for by study design?  *- Was consideration of confounding factors accounted for in study design?* | |  |  | |  | |  |
| 9 | | Are known confounders accounted for in the analyses? | |  |  | |  | |  |
| 10 | | Are the statistical tests used to assess the main outcomes appropriate?  *-Was there adequate adjustment for confounding in the analyses?*  *- Do the analyses adjust for different lengths of follow-up (if applicable)?* | |  |  | |  | |  |
| 11a | | Are the estimates reported with confidence intervals and in detail by sub-group (if appropriate)?  *- Were the findings reported clearly?* | |  |  | |  | |  |
| 11b | | Are statistically non-significant results presented? | |  |  | |  | |  |
| 11c | | Are data for relevant variables complete? | |  |  | |  | |  |
| 12 | | Was the conduct of the fieldwork appropriate to the study setting?  *-Was the allocation of the interviewer/interpreter sensitive to the background of the participant?*  *-Were fieldworkers trained and supported to work with people who have perpetrated domestic violence?* | |  |  | |  | |  |
| 13 | | Were ethical considerations appropriately considered?  -*Did researchers obtain informed consent from all participants?*  *- Did researchers take adequate precautions to safeguard participants’ anonymity and confidentiality?*  *-Did fieldworkers offer information about domestic violence support and referral options to all participants?*  -*Were fieldworkers appropriately trained to deal with participant distress?* | |  |  | |  | |  |
| 14 | | Do the findings support the conclusions? | |  |  | |  | |  |
| 15 | | Are the strengths and weaknesses of the research discussed? | |  |  | |  | |  |

Calculate total score (out of a possible total of 42):

1. Data from any identified mixed methods studies will be extracted and included in the quantitative review as appropriate. [↑](#footnote-ref-1)
